# Role of PAX8 in the regulation of MET and RON receptor tyrosine kinases in non-small cell lung cancer

**DOI:** 10.1186/1471-2407-14-185

**Published:** 2014-03-14

**Authors:** Rajani Kanteti, Essam El-Hashani, Immanuel Dhanasingh, Maria Tretiakova, Aliya N Husain, Sherven Sharma, Jay Sharma, Everett E Vokes, Ravi Salgia

**Affiliations:** 1 Department of Hematology/Oncology, University of Chicago Medical Center, Chicago, IL, USA; 2 Department of Pathology, University of Chicago Medical Center, Chicago, IL, USA; 3 Department of Medicine, University of California, Los Angeles, CA, USA; 4 Department of stem cell Research, Cellprogen Inc, San Pedro, CA, USA; 5 University of Chicago, Department of Medicine, 5841 S. Maryland Avenue, Chicago, IL 60637, USA

**Keywords:** PAX8, MET, RON, NSCLC

## Abstract

**Background:**

Non-small cell lung cancers (NSCLC) are highly heterogeneous at the molecular level and comprise 75% of all lung tumors. We have previously shown that the receptor tyrosine kinase (RTK) MET frequently suffers gain-of-function mutations that significantly promote lung tumorigenesis. Subsequent studies from our lab also revealed that PAX5 transcription factor is preferentially expressed in small cell lung cancer (SCLC) and promotes MET transcription. PAX8, however, is also expressed in NSCLC cell lines. We therefore investigated the role of PAX8 in NSCLC.

**Methods:**

Using IHC analysis, PAX8 protein expression was determined in archival NSCLC tumor tissues (n = 254). In order to study the effects of PAX8 knockdown on NSCLC cellular functions such as apoptosis and motility, siRNA against PAX8 was used. Confocal fluorescence microscopy was used to monitor the localization of MET, RON and PAX8. The combinatorial effect of PAX8 knockdown and MET inhibition using SU11274 was investigated in NSCLC cell viability assay.

**Results:**

Relative levels of PAX8 protein were elevated (≥ + 2 on a scale of 0–3) in adenocarcinoma (58/94), large cell carcinoma (50/85), squamous cell carcinoma (28/47), and metastatic NSCLC (17/28; lymph node). Utilizing early progenitors isolated from NSCLC cell lines and fresh tumor tissues, we observed robust overexpression of PAX8, MET, and RON. PAX8 knockdown A549 cells revealed abrogated PAX8 expression with a concomitant loss in MET and the related RON kinase expression. A dramatic colocalization between the active form of MET (also RON) and PAX8 upon challenging A549 cells with HGF was visualized. A similar colocalization of MET and EGL5 (PAX8 ortholog) proteins was found in embryos of *C. elegans*. Most importantly, knockdown of PAX8 in A549 cells resulted in enhanced apoptosis (~6 fold) and decreased cell motility (~45%), thereby making PAX8 a potential therapeutic target. However, the combinatorial approach of PAX8 knockdown and treatment with MET inhibitor, SU11274, had marginal additive effect on loss of NSCLC cell viability.

**Conclusion:**

PAX8 provides signals for growth and motility of NSCLC cells and is necessary for MET and RON expression. Further investigations are necessary to investigate the therapeutic potential of PA8 in NSCLC.

## Background

Lung cancer is a devastating illness with an overall survival of 17% in NSCLC. NSCLC has differential histological classification (adeno carcinoma, squamous cell carcinoma and large cell carcinoma) and prognosis is dependent considerably on the stage of the cancer at the time of diagnosis [1]. In order to make an impact on the disease, understanding key molecular changes is crucial. Most recently, EGFR mutations, ALK/ROS1 translocation and MET amplification have been shown in a subset of NSCLC [2]. A much wider impact, however, can be achieved by investigating the role of transcription factors, as they regulate gene transcription programs.

*PAX* genes comprise a relatively small family with 9 members that are highly conserved through evolution. They play key indispensable role in development. PAX proteins are defined by the presence of an 128 amino acid DNA binding domain at their amino terminal end referred to as the, ‘Paired Domain’, which makes sequence specific contact with DNA and regulates the transcription of select genes. *PAX* genes are divided into four different subgroups based on the presence or absence of additional domains such as homeodomain and octapeptide motif [3].

We have previously shown differential expression of PAX5 and PAX8 in lung cancer [4]. While PAX5 is selectively expressed in SCLC cells, the expression of PAX8 was found mostly in NSCLC cells. We have also shown that PAX5 positively regulates the transcription of MET in SCLC. We therefore investigated further the role of PAX8 in NSCLC. Under conditions of normal development, PAX8 is expressed in the thyroid, kidneys, some part of central nervous system, and the placenta. In adults it is expressed in thyroid follicular cells and is indispensable for the differentiation of thyroid cells [5]. In follicular thyroid carcinoma, PAX8 undergoes gene rearrangement as a result of (2;3) (q13;p25) chromosomal translocation with peroxisome proliferator-activated receptor- γ(*PPARγ)*[6]. Significant expression of PAX8 was found in most carcinomas of thyroid, ovary and placenta [7-10]. PAX8 is known to activate the transcription of *BCl2*, which is an anti-apoptotic gene, and it is also involved in suppression of *p53* thus suggesting a role in tumor initiation and progression [11,12].

We have previously shown that the simple soil nematode, *Caenorhabditis elegans,* can be used as a model to study the basic signaling pathways involved in lung cancer [13]. Their relatively short life cycle (~3 days), completely sequenced genome, invariant cell lineage make them attractive *in vivo* models. Our previous work demonstrated that the forced expression of a MET mutant, originally discovered in human NSCLC, results in an abnormal vulval phenotype with marked hyperplasia. In *C. elegans*, PAX8 (also 2 and 5) equivalent is EGL-38, which plays an indispensable role in the hermaphrodite egg laying process [14].

We show here that PAX8 is preferentially overexpressed in NSCLC tumors. In NSCLC cells, upon stimulation with HGF, we observed a strong nuclear colocalization of PAX8 and phosphorylated MET and RON in the nucleus and a similar colocalization was also seen in *C. elegans* eggs suggesting that this soil nematode can be used a model to study the genetics of MET/PAX8 and signaling axis. Silencing of PAX8 resulted in a significant decrease in not only PAX8 levels but also that of MET and RON expression. The functional consequences of loss of PAX8 expression were decreased viability and cell motility in NSCLC cells. Finally, treating PAX8 knockdown NSCLC cells with the MET small molecule inhibitor (SU11274) had no synergistic effect on the loss of cell viability. This is most likely due to the fact that PAX8 is essential for MET and RON expression.

## Methods

### Cell lines

NSCLC cell lines were obtained from the American Type Culture Collection (Manassas, VA) and were cultured in RPMI 1640 medium from Gibco/BRL supplemented with 10% (v/v) fetal bovine serum at 37°C with 5% CO2.

### Antibodies and other Reagents

PAX8 and PAX2 antibodies were purchased from Abcam (Cambridge, MA). The phospho-specific (pY1230/1234/1235) anti- MET rabbit polyclonal and total MET mouse antibody was from Invitrogen. EGFR, β Ron and p-Ron antibodies were purchased from Santa cruz Biotechnology (Santa Cruz, CA). SU11274 (3Z)-N-(3-Chlorophenyl)-3-(3,5-dimethyl-4-((4-methylpiperazin-1-yl)carbonyl)-1H-indole-5-sulfonamide, the MET small molecule inhibitor was from EMD Calbiochem (San Diego, CA). A set of four different small interfering RNAs (siRNAs) specific for PAX8 and scrambled control siRNA were purchased from Qiagen (Cambridge, MA). Recombinant human HGF was purchased from R & D systems (Minneapolis, MN).

### Immunoblotting

Whole cell lysates were prepared using RIPA lysis buffer (50 mM Tris (pH 8.0), 150 mM NaCl, 10% glycerol, 1%NP-40, 0.5% Sodium deoxycholate, 0.1% SDS) containing protease and phosphatase inhibitor cocktail. Protein concentrations were determined by using the Bradford Assay. Protein lysates about 80–100 ug were separated by 7.5% SDS-PAGE under reducing conditions and transferred to PVDF membranes (Millipore, Bedford, MA). The membranes were blocked in 5% BSA prepared in TBST. Proteins were detected by immunoblotting using kit from Boston Bioproducts (Worcester, MA).

### Transfection with PAX8 siRNA

A549 cells were plated in 60 mm plates at a density of 1.5 × 10^5^ in 10% RPMI and transfected with 100 nM siRNA or scrambled control RNA (scRNA) using oligofectamine transfection reagent according to the manufacture’s protocol (Life Technologies Grand Island, NY). After 96 h incubation with siRNA, the lysates were prepared using Ripa lysis buffer.

### Cell viability assay

For cell viability assay A549 cells were plated in 96 well tissue culture plates at a density of 1 × 10^4^ per well. Each experiment was done in 12 or more replicates. The next day, the cells were transfected with PAX8 and control siRNA as described above. Cell viability was determined using Alamar blue (Sigma, St. Louis, MO), a non-toxic compound, which gets reduced in the cell and emits fluorescence. The amount of reduced Alamar blue formed is proportional to metabolic activity of cells. After 96 h, the cells were washed with PBS and 100 μl fresh growth medium was added. Alamar Blue was added to the media to get final concentration 10%. Plates were incubated at 37°C for 3 to 8 hours and fluorescence was measured using a plate reader (530/590 ex/em).

### Apoptosis assay

The A549 cells were plated in 60 mm plates and PAX8 was knocked down using PAX8 specific siRNA. The percentage of apoptosis was determined by Annexin V staining using FITC Apoptosis detection kit from BD Biosciences (San Jose, CA) according to the manufacturer’s instructions.

### Wound healing assay

PAX8 knock down A549 cells were trypsinized. 1 × 10^5^ cells were replated in 24 well plates containing cell culture inserts (IBIDI, Verona, WI). The next day, the inserts were removed and the cells were washed with PBS and fresh media was added. The fine scratch created by the inserts was photographed at various time points.

### Migration assay

Cell migration was determined in A549 cells transfected with PAX8 siRNA or scrambled siRNA. After a 72 h transfection, cells were trypsinized and replated for migration assay into Transwell chambers (BD Biosciences, San Jose, CA) containing 500 μl serum free media at a density of 1 × 10^5^ cells per chamber. The chambers were placed into wells of 24 well plate containing 500 μl RPMI with 10% FBS as a chemo attractant. After 24 h, the top and bottom chambers were washed twice with PBS. The cells were then fixed in 4% paraformaldehyde for 10 min at room temperature and washed again with PBS. After removing non-migrated cells from the top chamber using a cotton swab, the remaining cells were stained with Crystal violet for 30 minutes and then rinsed thoroughly with distilled water to remove the extra stain and dried overnight. Images of each chamber were captured using a microscope and the migrated cells were counted using Image J.

### Tissue microarray construction and immunohistochemistry

The procedure is similar to the one we previously used [15]. Tissue blocks of patients with lung cancer at the University of Chicago Medical Center (diagnosed between 1992 and 2005) were selected for the study after obtaining appropriate institution IRB approval. Clinical and pathological information were collected and the database tabulated in an anonymous fashion. Samples of tissue sections from primary tumors were identified and 1.5-mm cores of the identified tissues were punched from the donor blocks and inserted into a recipient block. Where available, the edge of primary tumor and corresponding normal lung tissue were identified and included into the tissue microarray (TMA). The TMA was cut in 5-μm sections and immunohistochemistry was performed as detailed below. The TMA slides were deparaffinized in xylene and rehydrated through graded ethanol solutions to distilled water and then washed in Tris-buffered saline (TBS). Antigen retrieval was carried out by heating sections in Citrate Buffer (pH = 6) for 15 min in a microwave oven. Endogenous peroxidase activity was quenched by incubation in 3% H2O2 in methanol for 5 min. Non-specific binding sites were blocked using Protein Block (DAKO) for 20 min. Then tissue sections were incubated for 1 h at room temperature with PAX8 Goat antibody from Abcam. This step was followed by 30 min incubation with goat anti-mouse IgG conjugated to a horseradish peroxidase (HRP)-labeled polymer (ImmunoDetector HRP, CA). Slides were then developed for 5 min with 3-3’-diaminobenzidine (DAB) chromogen, counterstained with hematoxylin, and coverslipped. Negative controls were performed by substituting primary antibody step with non-immune mouse immunoglobulins. Cytoplasmic and membranous staining for each sample was quantified manually using a conventional four-point pathology scoring system (0 - no staining, 1 - weak staining, 2 - moderate staining and 3 - strong staining), and then by an Automated Cellular Imaging System (ACIS, Chromavision, USA). These parallel methods were found to have a strong positive correlation with //r2 = 0.75// (p < 0.0001). While similar trends were seen in manual scoring and ACIS analyses, we report only ACIS data.

Automated measurement of immunostaining intensity based on three related color parameters: the color defined by hue, the “darkness” defined as luminosity, and density of the color defined as the saturation. ACIS software was programmed by experienced user-pathologist (MT), by setting the color-specific thresholds, to determine the intensity of brown (cytoplasmic and membranous) positivity of cells within the outlined areas of interest. For each TMA spot we selected representative areas of tumor containing comparable number of cells (approximately 200–500 cells). ACIS software calculated the average intensity for each region as a measure of IOD (integrated optical density) in cytoplasmic and membranous compartments. The IOD of each image (region) is given as the average of optical densities of each molecule (pixel) within the region. Computing of IOD is directly proportional to the concentration of molecule recognized by the stain according to Beer-Lambert Law. IOD is a proxy for antigen content and it is calculated as intensity multiplied by brown area in microns. For comparison purposes we normalized IOD value to the entire measured area by calculating //IOD/10 μm^2//.

### Immunofluroscence and confocal microscopy

A549 cells were cultured in medium lacking FBS on glass coverslips coated with poly-lysine in 6 well culture plate overnight. The cells were then stimulated with HGF (100 ng/ml) for 10 min. The cells were fixed and permeabilized as previously reported [4] and incubated with primary antibodies against p-MET, or p-RON, or PAX8 and appropriate secondary antibodies labeled with fluorescein (CY3 and CY5) and the samples were mounted in vectashield.

Immunostaining of *C. elegans* embryos was performed as described [16]. Wild type N2 adult worms were placed on poly-lysine coated slides. A coverslip was overlaid, and pressure was applied to extrude the embryos. The slides were placed on dry ice or liquid N2 and coverslips were immediately removed. The worms were fixed at -20°C in methanol for 5 min and then incubated for 30 min in PBST (PBS containing 0.1% Tween 20). Worms were then washed once with PBST, incubated in 5% nonfat dry milk, for 1 hour, washed once again with PBST and incubated overnight at 4°C with primary antibodies. Next day the slides were washed three times for 10 min with PBST and then incubated with Alexafluor secondary antibodies for 1 h at room temperature. Slides were washed once again three times with PBST and mounted using mounting media containing Dapi.

### Statistical analysis

Results for viability, wound healing, and migration assays were expressed as mean ± SE. The statistical significance between the means was measured by t-test.

## Results

### PAX8 protein is relatively highly expressed in a variety of NSCLCs including their metastatic forms

**Figure 1:**
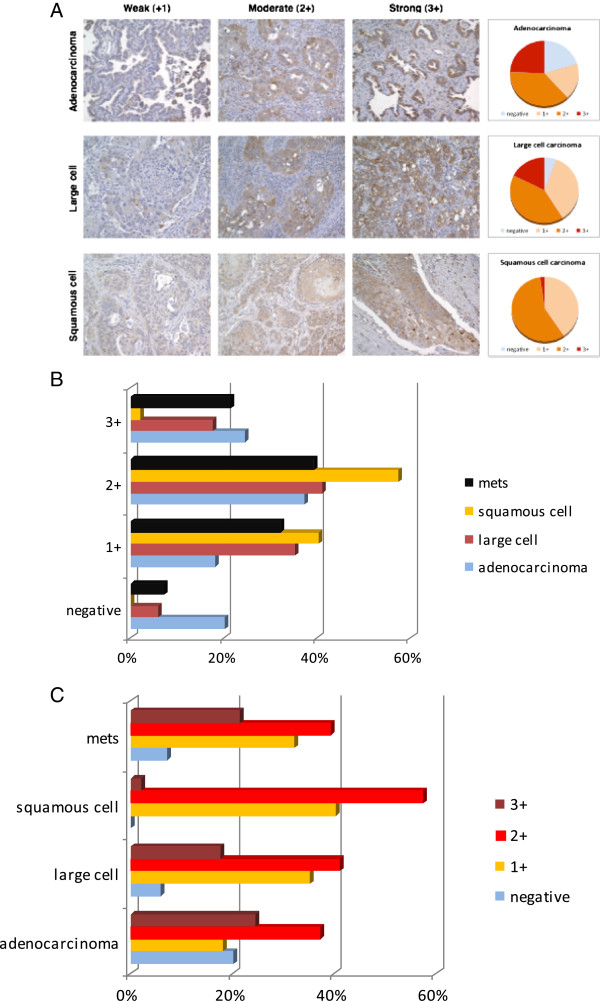
**PAX8 protein expression in NSCLC tumor tissues. A**. PAX8 staining was carried out as described in Methods. Representative immunohistochemistry pictures of weak (+1), moderate (+2) and strong (+3) of PAX8 expression in adeno (top panel), large cell (middle) and squamous cell (lower) carcinoma of the lung are shown. To the right of each panel, pie charts that represent PAX8 expression intensity in that particular type of lung cancer is shown. **B**. The bar graph summarizes the percentage NSCLC tumors that express various amounts of PAX8 protein. **C**. Intensity of PAX8 protein expression in adeno, large cell and squamous NSCLC tumors is shown.

We have previously shown that PAX8 is preferentially expressed in a variety of NSCLC cell lines [4]. In order to validate the expression of PAX8 in NSCLC tumors, we determined PAX8 protein expression using TMA (Tissue Microarray) which represents archival NSCLC tumor tissues and carried out IHC analysis of PAX8 expression in adeno carcinoma (n = 94), Large cell carcinoma (85), squamous cell carcinoma (47), and metastatic tumor tissue samples (28) are shown in Figure 1. The various panels in Figure 1A represent PAX8 protein expression in adeno, large cell, and squamous carcinomas, with representative images of increasing intensities from left to right. The pie charts to the right show the relative proportions of weak, moderate, and strong staining in the three types of NSCLC. The bar graphs in Figure 1B represent the above results as percentages and the detailed data is summarized in Table 1. The intensity of PAX8 expression increased from squamous cell carcinoma to large cell carcinoma to adenocarcinoma. The highest % of negative cases was found in adeno carcinoma (Figure 1B-C and Table 1).

**Table 1:** PAX8 expression in various NSCLC tumors as evidenced from IHC analysis

**Tumor type**	**0**	**1+**	**2+**	**3+**	**N case**
Adenocarcinoma	19	17	35	23	94
Large carcinoma	5	30	35	15	85
Squamous cell carcinoma	0	19	27	1	47
Metastatic	2	9	11	6	28

The intensity of PAX8 expression scored for various NSCLC archival tumors examined are shown, with 3+ being the maximum. The total number of samples analyzed for each category is shown in the last column.

In adeno carcinoma, almost 20% cases were PAX8- nonexpressors, whereas in large cell carcinoma, only 6-7% of the cases were negative for PAX8 expression. The 47 squamous cell carcinoma tissues examined, however, were all positive for PAX8 protein expression. No major differences were noted with respect to PAX8 expression when primary tumors were compared with metastatic tumor samples (Figure 1B-C and Table 1).

### PAX8 promotes expression of MET and RON receptor tyrosine kinases

**Figure 2:**
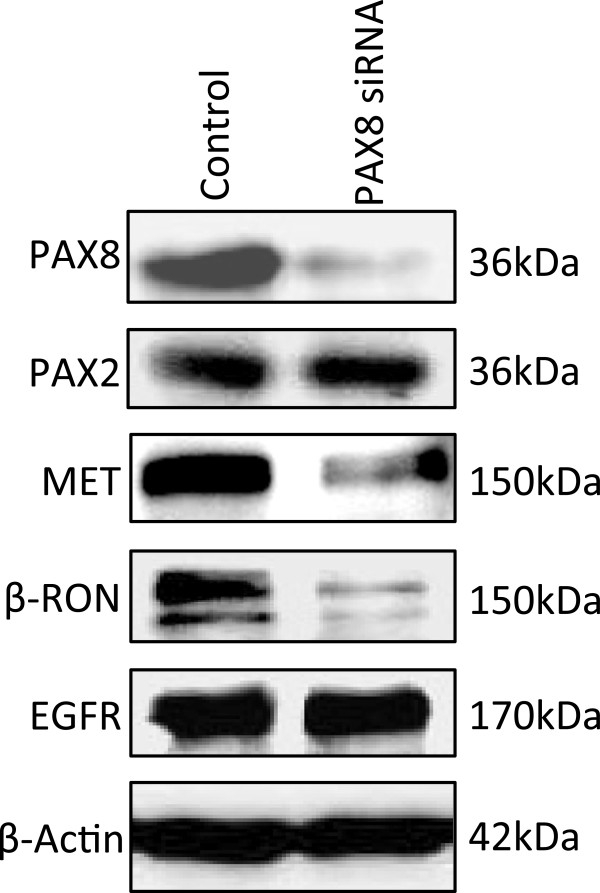
**Effect of PAX8 knockdown on the expression of PAX8, PAX2, MET and RON in A549 cells.** A549 cells were transfected with scrambled and PAX8 specific siRNA at 100nM concentration. The whole cell lysates were prepared and then immunoblotted with anti-PAX8, anti-PAX2 antibodies. The lysates were used in parallel to determine the MET, RON, and EGFR expressions using specific antibodies in immunoblotting. β-actin was used as a loading control. As shown, the knockdown of PAX8 had no effect on PAX2 levels. However a precipitous decrease in both RON and MET expression levels was observed.

We previously showed that PAX5 was a key transcription factor for MET transcription [4]. In order to check the relationship between PAX8 and MET/RON levels, we knocked down PAX8 expression using specific siRNA and observed the effect on the levels of other PAX transcription factors and MET and RON expression levels. As shown in Figure 2, there was a dramatic loss in PAX8 expression only when the NSCLC cells were treated with PAX8 specific siRNA but not with control scrambled siRNA. The specificity of PAX8 siRNA can be appreciated by the unaltered levels of PAX2 protein. Loss of PAX8 expression was also accompanied by a dramatic decrease in the expression of both MET and RON but not EGFR tyrosine kinases. This suggests that PAX8 is a key transcription factor for MET and RON, as was the case with PAX5.

### Colocalization of PAX8 and activated MET (p-MET)

**Figure 3:**
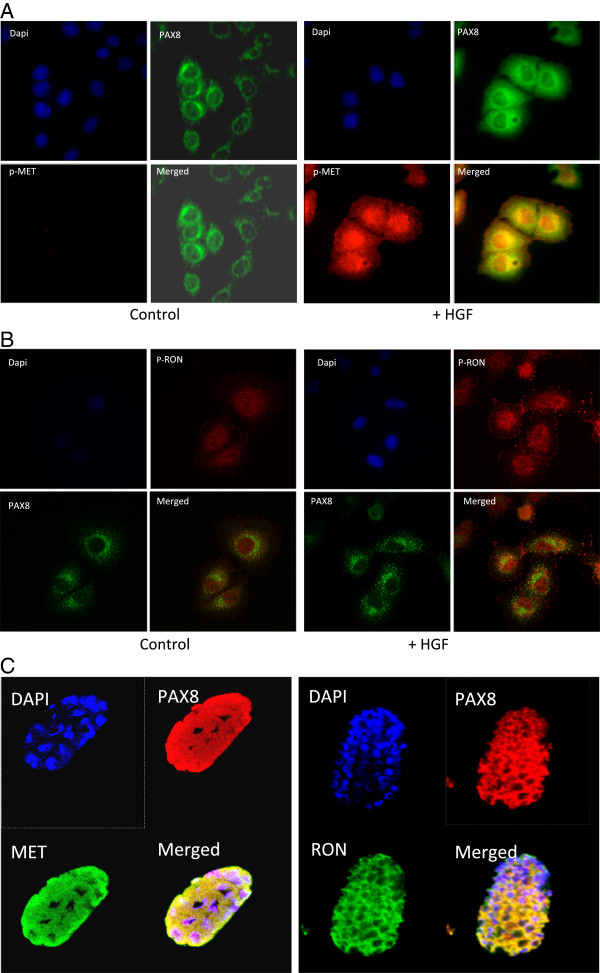
**MET and RON are localized to the same cellular compartment that is evolutionarily conserved. A**. The colocalization of phospho-MET (active form of MET) with PAX8 transcription factor in the peri-nuclear area with HGF stimulation. Representative confocal pictures showing individual staining patterns for phospho–MET and PAX8 and the two merged in control (left panel) and HGF treated (right panel) in A549 cells are shown. The cells were treated with HGF for 15 min at concentration of 100 ng/ml. **B**. The colocalization of phospho-RON (active form of RON) with PAX8 transcription factor in the peri-nuclear area with HGF stimulation: Representative confocal pictures revealing staining patterns of p-RON and PAX8 as well as their merged pictures are shown. **C**: Colocalization of PAX8 with MET and RON in *C. elegan’s* embryos. Immunofluorescence of *C. elegans* was performed as described in Methods. The left panel shows representative confocal pictures of *C. elegan’s* embryos stained with DAPI, PAX8, MET and merged. The right side panel shows staining with DAPI, PAX8, RON, and merged.

Earlier, we showed that PAX5 not only promoted MET transcription but also colocalized with p-MET (active MET) in the nucleus, in SCLC cells. We therefore checked whether PAX8 also behaved in a similar fashion in NSCLC cells. The representative confocal images of PAX8 and p-MET in resting and HGF stimulated NSCLC cells are shown in Figure 3A. In untreated cells, PAX8 expression was mostly restricted to the perinuclear region and p-MET was hardly seen. However, in HGF treated cells, there was a sharp increase in the p-MET expression, most of which was localized to the nucleus. Also there was a strong colocalization of PAX8 and p-MET in the nucleus, suggesting that they could be functionally linked. A similar colocalization between MET related kinase RON and PAX8 was observed (Figure 3B). In addition, we also observed a strong colocalization of PAX8 (EGL5) with MET and RON in *C. elegans* embryos (Figure 3C), suggesting that the highly conserved association between MET, RON and PAX8 could have strong functional significance.

### PAX8 knockdown induces Apoptosis in A549 cells

**Figure 4:**
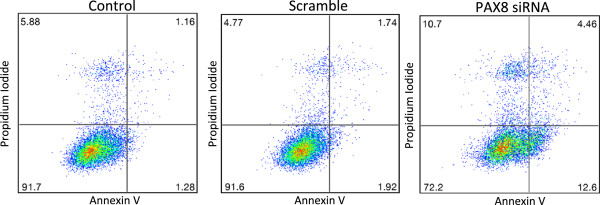
**Effect of PAX8 knockdown on A549 NSCLC cell apoptosis.** A549 cells were transfected with control (scramble) and PAX8 specific siRNA at 100 nM concentration for 72 h. Apoptosis was quantified using flow cytometry after staining with Annexin V/PI and effect of loss of PAX8 expression on apoptosis is shown in the representative charts. Both early (right lower quadrant) and late (right upper quadrant) stage apoptosis increased in PAX8 knockdown cells as shown by flow cytometry analysis.

The effect of PAX8 knock down in A549 cells on apoptosis was investigated using flow cytometry based analysis after staining with Annexin V and PI and representative results are shown in Figure 4. There was a six fold increase in early apoptosis (compare bottom right quadrants in scrambled and PAX8 siRNA panel) thereby indicating a cell survival role for PAX8.

### PAX8 promotes cell migration in NSCLC cells

**Figure 5:**
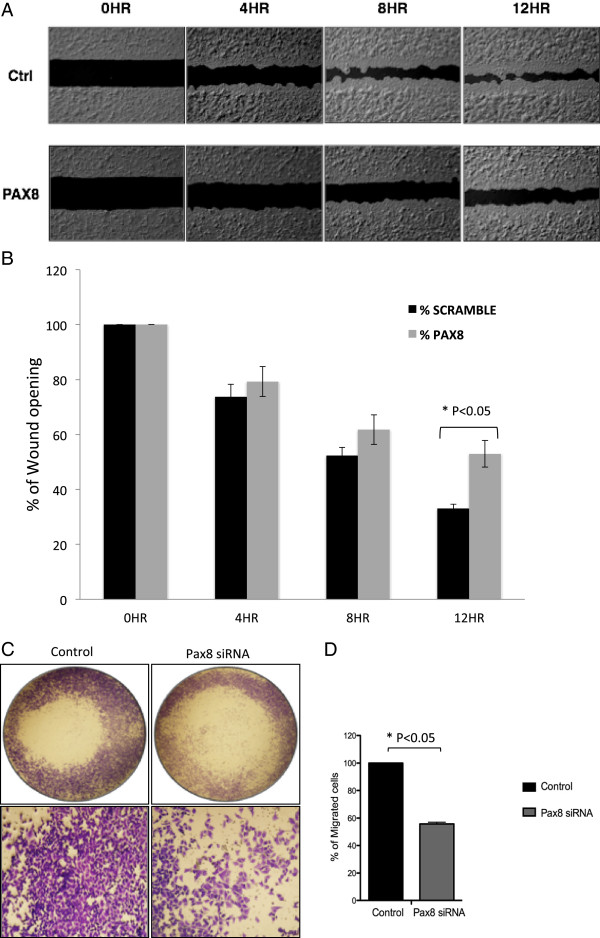
**Effect of PAX8 knock down on A549 NSCLC cell motility and migration. A**. Wound healing assay was performed in A549 cells transfected with control (scramble) and PAX8 specific siRNA for 72 h and then replated in 24 well plate containing cell culture inserts. The fine scratch created by the inserts was monitored and photographed for 12 hr. Representative pictures of the degree of wound closure in A549 cells when subjected to a scratch in control and PAX8 knock down cells at 12 hr. **B**. The open wound at each time point was quantified and normalized to 0 h. The experiments were done in triplicate and the average data is shown with standard error bars. **C**. A549 cells were transfected with either control or PAX8 specific siRNA as described in Methods. After 96 h the cells were replated in Boyden chambers for migration assay. Migrated cells were fixed and stained with Crystal violet. Representative images of control and PAX8 knockdown cells were captured using a microscope. **D**. The migrated cells were counted using Image J. The experiments were done in triplicate and the averages with standard error bars are shown.

Next we determined the effect of PAX8 knockdown on wound healing, which is routinely used to measure the cell motility - an index of metastatic potential. A549 cells were treated with either control (scrambled) or PAX8 specific siRNA, allowed to grow to confluence in 24 well plates with cell culture inserts, and then inserts were removed to create a fine scratch. The closure was documented over a period of 12 h as shown in Figure 5A. There was a clear loss in cell motility as reflected by prominent gaps in the scratches made in PAX8 knock down plate compared to control cell monolayer. The quantitative differences between control and PAX8 knockdown cells are shown as bar graphs in Figure 5B.

We also carried out a cell migration assay using Boyden Chamber with fetal calf serum as chemoattractant. The dramatic decrease in cell migration in PAX8 knockdown cells compared to control cells can be appreciated from representative photographs shown in Figure 5C and the summary results shown as bar graphs in Figure 5D.

### Combinatorial effect of MET inhibitor SU11274 and knock down of PAX8 in NSCLC cells has an additive effect on the loss of cell viability

**Figure 6:**
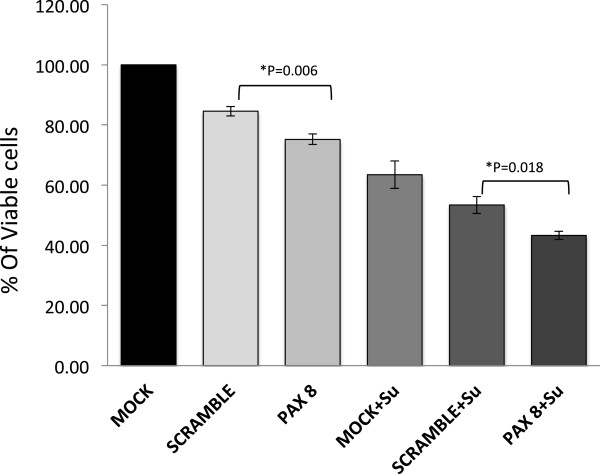
**The Combinatorial effect of PAX8 knockdown with MET inhibitor (SU11724) on NSCLC cells.** A549 cells were transfected with either control or PAX8 specific siRNA. As shown they were then treated with SU11274. The viability assay was done using Alamar Blue. The histograms represent percentage of cell viability with standard error bars.

The knock down of PAX8 had a significant but marginal decrease in cell viability compared to controls (compare 75% Vs. 83%%). Treatment of A549 cells with small molecule inhibitor of MET, SU11274, however resulted in an almost 50% loss in cell viability that further decreased to 43% in PAX8 knocked down cells under comparable conditions. This suggests that the combinatorial effect is additive and not synergetic (Figure 6).

## Discussion

PAX transcription factors usually play an indispensable role in various developmental processes. For instance PAX5 plays an important role in B cell development and PAX8 has a key role in the differentiation of thyroid epithelium [17]. However, we have previously shown that PAX5 and PAX8 are differentially expressed in lung cancers; while PAX5 expression is restricted to SCLC cells, PAX8 expression is apparent more in NSCLC cell lines [4]. Such out-of-context expression underscores the loss of any normality in lung cancer. In this paper, we have shown that PAX8 is functional in NSCLC and is overexpressed in a majority of NSCLCs, irrespective of their sub-type, including their lymph node metastatic forms. As with PAX5, there appears to be strong physical as well as a functional link between PAX8 and the receptor tyrosine kinases MET and RON. A strong cellular colocalization between PAX8 and MET was observed and the loss of PAX8 expression in NSCLC cells also revealed a concomitant loss in the expression of MET and RON receptor kinases but not EGFR. With respect to functionality, PAX8 is very likely to significantly contribute to both lung tumor growth and metastasis as surmised from its role in cell viability and motility. Knockdown of PAX8 resulted in a significant but modest loss in cell viability, and further treatment with the MET inhibitor SU11274 had only a marginal additive effect.

Under physiological conditions, PAX8 is required for the normal development of the kidneys, thyroid gland, and the Mullerian system [13,18,19]. Recently, a systematic study of more than 1100 (normal and tumor) cases revealed that it was overexpressed in majority of the renal, ovarian, and endometrial cancers; whereas only one out of 100 lung cases turned out to be positive [20]. This is clearly in contrast to the findings reported here. Our IHC analysis of archival lung tumor tissues unmistakably showed that the majority of large cell carcinoma (94%), adeno carcinoma (80%), squamous cell carcinoma (100%), and metastatic tumors in lymph nodes (93%) were positive for PAX8 expression (n = 254). Also, overexpression of PAX8 in endometrial cancer is associated with a poor prognosis [19,21]. Although site directed loss-of-function mutants have been reported in hypothyroidism, no gain-of-function mutants have been reported for PAX8 in any cancers. Using genomic DNA extracts prepared from NSCLC archival tumor tissues, we failed to see any mutations in PAX8 genomic DNA (exons), while MET mutations were apparent (data not shown). In 36% of follicular thyroid cancers, there is a chromosomal translocation (2;3) (q13;p25) resulting in the formation of PAX8-PPAR-γ (peroxisome proliferator activated receptor-γ) fusion protein [6]. While, in vitro experiments have clearly demonstrated the oncogenecity of the above fusion protein, recent studies in follicular thyroid carcinoma suggest a better prognosis in cases where the fusion protein was found to be overexpressed [22].

The silencing of PAX8 in NSCLC cells not only resulted in decreased levels of MET, but also that of RON. MET and RON are family members of the receptor tyrosine kinase and are important in the pathogenesis of a number of malignancies. The synergistic role of MET/RON is just beginning to be defined and their inhibition appears to be more important than inhibition of either one of the receptors. The findings here show that MET/RON could potentially be down regulated by decreasing expression by the PAX8. A recent PAX8 silencing study using rat FLT-6 thyroid cells revealed a total of 2815 genes modulated. A key finding of this study was that PAX8 is the primary regulator of thyroid morphogenesis and differentiation. In addition to genes that play a direct role in thyroid development and function, the authors identified several genes regulated by PAX8 that belonged to cell proliferation, apoptosis, tyrosine kinases, DNA replication, and anion transport. One of the genes positively regulated by PAX8 was MET. However the decrease in MET transcripts in PAX8 knockdown cells, although significant, was only 1.4 fold [23]. In the present study, we observed a dramatic decrease in the protein expression levels of MET and RON. We previously showed the presence of a functional PAX binding site in MET (and also RON) proximal promoter region [4]. It thus appears that PAX8 regulates the transcription of MET and RON but not EGFR. Similar to our earlier report with respect to PAX5 [4], we also observed a strong colocalization of PAX8 with active forms of MET and RON kinases, especially after the NSCLC cells were stimulated with HGF. The fact that the colocalization between PAX8 and the RTKs is conserved even in the simple nematode suggests a strong functional link. One possibility that is being currently tested is whether the active forms of MET and RON as feedback inhibitors of PAX8 transcription.

We have shown here clearly that PAX8 promotes cell motility and wound healing, a harbinger of its role in metastasis. Most likely, the above effect is through its ability to promote the transcription of both MET and RON RTKs. We and others showed that both MET and RON provide essential growth signals for lung tumor development and also play a key role in metastasis [4]. It is not surprising that PAX8 knockdown results in significant loss in A549 cell viability; as such a role was demonstrated for PAX transcription factors including PAX8 in the promotion of cancer cell growth [12,24,25]. In order to rule out the vagaries of using one transformed cell line, A549, we also repeated the functional assays using H1993, another NSCLC cell line and investigated the effects of PAX 8 knockdown on loss of cell motility and viability. We have consistently observed that they were similar to that shown for A549 cells. For instance, knockdown of PAX8 in H1993 cells resulted in a loss in the ability to close the gap created in scratch assay by about 20% at 12 h that was comparable to that seen in PAX8 knockdown A549 cells in relation to respective scrambled RNA treated controls, although actual values were somewhat different. In terms of cell viability, PAX8 knockdown had a marginal inhibitory effect in both A549 and H1993 cells. Inhibition of MET using specific inhibitors is also known to adversely affect NSCLC cell viability and is the basis for several ongoing clinical trials aimed at testing the efficacy of MET inhibitors in NSCLC and other cancers [26]. Our results using SU11274, an ATP-competitive small molecule inhibitor of the catalytic activity of MET in A549 cells, clearly support the above. However, a combinatorial approach of using SU11274 in PAX8 knockdown background failed to induce synergetic loss in A549 cell viability. Also in H1993 cells, it was the MET inhibitor (SU11274) that had maximum inhibitory effect on cell viability that was comparable to that observed in A549 cells. There was however no discernible synergy on loss of cell viability when the SU11274 was used in PAX8 knockdown H1993 cells. These observations substantiate our contention that PAX8 knockdown effects in NSCLC cells are consistent and due to the use of any select NSCLC cell line. The lack of synergy can be logically explained by the fact that a loss in PAX8 transcription factor activity results in a dramatic loss in MET expression, thereby making the use of SU11274 redundant, at least with respect to MET. Synergy in killing NSCLC is more likely to be achieved by combining PAX8 knockdown with a non-overlapping disparate approach such as cisplatin that forms DNA adducts [27].

## Conclusion

In conclusion, we report here a potential therapeutic target PAX8 whose silencing in NSCLC cells promotes loss in viability and motility; most likely through the deprivation of essential signals from MET and RON RTKs.

## Competing interests

The authors declare that they have no competing interests.

## Authors’ contribution

**RS** and **RK** conceived and designed the study. **RK, EEH** and **ID** performed the experiments and assembled the data. **RS** and **RK** analyzed the data and prepared the manuscript. **RS** supported the study financially. **MT** and **AH** did the IHC analysis. **SS** performed the immunoblot analysis. **JS** performed the characterization of cell lines. **EV** obtained the tumor and tissues with clinical information where pertained. All authors contributed extensively to the work presented in this paper. All authors read and approved the final manuscript.

## Pre-publication history

The pre-publication history for this paper can be accessed here:

http://www.biomedcentral.com/1471-2407/14/185/prepub
